# Chronic constriction injury-induced microRNA-146a-5p alleviates neuropathic pain through suppression of IRAK1/TRAF6 signaling pathway

**DOI:** 10.1186/s12974-018-1215-4

**Published:** 2018-06-09

**Authors:** Zhiyao Wang, Fan Liu, Min Wei, Yue Qiu, Chao Ma, Le Shen, Yuguang Huang

**Affiliations:** 1 Department of Anesthesiology, Peking Union Medical College Hospital, Chinese Academy of Medical Sciences, Peking Union Medical College, Beijing, 100730 China; 2 Institute of Basic Medical Sciences Chinese Academy of Medical Sciences, Department of Human Anatomy, Histology and Embryology, Neuroscience Center, School of Basic Medicine, Joint Laboratory of Anesthesia and Pain, Peking Union Medical College, No. 5 DongDanSanTiao, DongChengQu, Beijing, 100005 China; 3 Department of Anesthesiology, Zhongshan Hospital, Fudan University, Shanghai, 200032 China

**Keywords:** miRNA-146a-5p, TRAF6, IRAK1, Dorsal root ganglion, Spinal dorsal horn, Neuropathic pain

## Abstract

**Background:**

microRNA-146a-5p (miRNA-146a-5p) is a key molecule in the negative regulation pathway of TLRs and IL-1 receptor (TIR) signaling. Our recent study demonstrated that MyD88-dependent signaling pathway of TIR in the dorsal root ganglion (DRG) and spinal dorsal horn (SDH) plays a role in peripheral nerve injury-induced neuropathic pain. However, it was not clear whether and how miRNA-146a-5p regulates the TIR pathway of DRG and SDH in the development of neuropathic pain.

**Methods:**

The sciatic nerve chronic constriction injury (CCI) model of rat was used to induce chronic neuropathic pain. The levels and cellular distribution of miRNA-146a-5p were detected with quantitative real-time PCR (qPCR) and fluorescent in situ hybridization (FISH). The RNA level, protein level, and cellular distribution of IRAK1 and TRAF6 that is targeted by miRNA-146a-5p were detected with qPCR, western blot, and immunofluorescent. The pain-related behavioral effect of miRNA-146a-5p was accessed after intrathecal administration. Mechanical stimuli and radiant heat were used to evaluate mechanical allodynia and thermal hyperalgesia.

**Results:**

We found that the level of miRNA-146a-5p significantly increased in L4-L6 DRGs and SDH after CCI surgery; meanwhile, the protein level of IRAK1 and TRAF6 in DRGs was significantly increased after CCI. Intrathecal injection of miR146a-5p agomir or miRNA-146a-5p antagomir regulates miRNA-146a-5p level of L4-L6 DRGs and SDH. We found that intrathecal injection of miR146a-5p agomir can alleviate mechanical and thermal hyperalgesia in CCI rats and reverse the upregulation of IRAK1 and TRAF6 of L4-L6 DRGs and SDH induced by CCI. We furthermore found that intrathecal injection of miRNA-146a-5p antagomir can exacerbate the mechanical and thermal pain-related behavior of CCI rats and meanwhile increase IRAK1 and TRAF6 of L4-L6 DRGs and SDH expression even further.

**Conclusions:**

miRNA-146a-5p of DRG and SDH can modulate the development of CCI-induced neuropathic pain through inhibition of IRAK1 and TRAF6 in the TIR signaling pathway. Hence, miRNA-146a-5p may serve as a potential therapeutic target for neuropathic pain.

**Electronic supplementary material:**

The online version of this article (10.1186/s12974-018-1215-4) contains supplementary material, which is available to authorized users.

## Background

Neuropathic pain is a rather stubborn pain induced by nerve injury. It can persist for months to years, even after the primary injury has healed [1]. Many studies focus on the molecular mechanisms are related to neuropathic pain. However, there is no medication currently available that treat neuropathic pain in a complete and definitive way. Accumulating evidence demonstrates that neuroinflammation in the peripheral and central nervous system (e.g., dorsal root ganglion (DRG) and spinal dorsal horn (SDH)) is involved in peripheral nerve injury-induced neuropathic pain [2–4]. DRG neurons are responsible for the complication of neuropathic pain as they include mechanoceptor, thermoceptor, and pruritic sensor [2]. Peripheral nerve injury activates nociceptive pathways and alters gene expression in DRG neurons, which may contribute to the development and maintenance of neuropathic pain.

Recent studies describe immune-related proteins of DRG and SDH are key players for peripheral and central sensitization of neuropathic pain [5–8]. Toll/interleukin-1 receptors (TIRs) such as TLR4 and IL-1R are found not only expressed on immune cells but also on sensory neurons in DRGs and glial cells (microglia and astrocytes) in the SDH [9–14]. Targeting toll-like receptors (TLRs) such as TLR4 expressed on spinal glial cells has been reported to relieve mice neuropathic pain [5]. Our recent studies show that suppression of myeloid differentiation factor-88 adaptor protein (MyD88)-dependent signaling alleviates neuropathic pain induced by peripheral nerve injury in the rat [15]. The MyD88 is involved in TIRs, mediates activation of TIRs, leads to the NF-κB activation, and induces proinflammatory mediators [9, 16]. TIRs and its signaling pathway play important roles in the pathogenesis of neuropathic pain. The activation of TIRs also needs to recruit interleukin-1 receptor-associated kinase 1 (IRAK1) and tumor necrosis factor receptor-associated factor 6 (TRAF6) to activate NF-κB signaling pathway [16].

Recent studies found the activation of NF-κB, and binding the promoters NF-κB-sensitive genes induce transcription of hundreds genes including NF-κB-dependent miRNAs such as miRNA146a-5p [17, 18]. miRNA is a family of small endogenous non-coding RNA molecules that silence target mRNAs by binding to their 3′UTRs. The miRNAs of the DRG participate in nociceptive modulation in the somatosensory pain [19]. miRNAs affect neuropathic pain by regulating key proteins in the pain progress, resulting in hyperalgesia and allodynia [20]. Mounting evidence suggests that miRNA-146a-5p is involved in the innate immune response and can reduce inflammation by targeting both TRAF6 and IRAK1 in monocytes, macrophages, and astrocytes [21–24]. Previous research demonstrated that spinal miRNA-146a could contribute to osteoarthritic pain of knee joints [25]. Also, Lu et al. found that miRNA-146a of astrocytes could attenuate SNL-induced neuropathic pain by suppressing TRAF6 signaling in the spinal cord [26]. However, the role of miRNA-146a-5p in DRG and SDH of nerve injury-induced neuropathic pain has not been fully investigated. How miRNA-146a-5p modulates the downstream target gene of DRG neurons in chronic constriction injury (CCI) is still unknown. TRAF6 and IRAK1 of TIR signaling may play an important role for neuroinflammation in DRG neurons of CCI model.

In the current study, we evaluated the expression of miRNA-146a-5p and its target genes, namely, IRAK1 and TRAF6, in the DRG of rats with CCI. We also intrathecally administered miRNA-146a-5p agonist (miRNA-146a-5p agomir) or antagonist (miRNA-146a-5p antagomir) to investigate the function of miRNA-146a-5p in modulating neuropathic pain. Our data demonstrated that miRNA-146a-5p can alleviate CCI-induced mechanical and thermal hyperalgesia through inhibition of IRAK1 and TRAF6 and may be the target for protection against chronic pain.

## Methods

### Animals

Male Sprague-Dawley (SD) rats weighing 200–250 g were acquired from Laboratory Animal Center of Peking Union Medical College Hospital, Chinese Academy of Medical Sciences. Animals were randomly assigned to treatment or control groups. These rats were bred in a specific pathogen-free environment in 12-h light-dark cycle. The rats were fed with rodent diet and water. These experiments were approved by the Institutional Animal Care and Use Committee in Chinese Academy of Medical Sciences.

### Rat model of neuropathic pain

In accordance with the study of Bennett and Xie YK [27], we performed CCI on rats anesthetized through intraperitoneal injection of sodium pentobarbital (40 mg/kg) under aseptic condition. After the sciatic nerve of the mid-thigh level on each side was exposed, four snug ligatures of chromic gut suture were loosely tied around the nerve with about 1-mm space between the knots. The sciatic nerves of sham animals were exposed without ligation.

### Behavioral test

Eight rats were included in each group. Paw withdrawal threshold (PWT) in response to mechanical stimuli was used to access mechanical allodynia by using Von Frey filaments 1 day before operation and 1, 3, 5, 7, 14, and 21 days after the operation. Paw withdrawal latency (PWL) in response to radiant heat was used to evaluate thermal hyperalgesia. Three repeat measures were performed in each rat with a 5-min interval. This test was performed at 10 a.m. on day 1 preoperation and days 1, 3, 5, 7, 14, and 21 postoperation. At the end of behavior testing, the L4-L6 DRGs and SDH were chronologically harvested and rapidly frozen at − 80 °C.

### Intrathecal catheter implantation and intrathecal injection

Eight rats were included in each group. A PE10 catheter (length, 15 cm) was intrathecally implanted using a previously described technique [28, 29]. Briefly, rats were intraperitoneally anesthetized with 10% chloral hydrate (300 mg/kg). A partial laminectomy at L5/L6 was performed to position the intrathecal catheter, and the dural membrane was exposed. The catheter was inserted through a dural incision and passed by 2 cm into the intrathecal space. The catheter was secured with 4/0 silk threads to the bones and muscles. After implantation, all rats were allowed to recover for a minimum of 2 days prior to the experiments. Rats presenting motor weakness or signs of paresis upon recovery from anesthesia were killed. Proper location of the catheter was confirmed through hind limb paralysis after injection of 10 μL of 2% lidocaine.

Intrathecal drug was administered with a microinjection syringe connected to the intrathecal catheter. CCI rats were randomly divided for intrathecal injecting miRNA-146a-5p agomir (Ribobio, China), agomir control (Ribobio, China), miRNA-146a-5p anatagomir (Ribobio, China), or antagomir control (Ribobio, China). Each drug (5 nmol) was intrathecally administered in 20 μL volume on the surgery day and on days 4, 8, and 12 after CCI surgery.

### Quantitative real-time PCR

Total RNA was isolated with TRIzol reagent (Invitrogen Life Technologies) and reverse-transcribed using a reaction mixture in accordance to the manufacturer’s instruction. RNA quality and quantity were determined with a NanoDrop spectrophotometer (ND-1000; NanoDrop Technologies), and RNA integrity was assessed through gel electrophoresis. Quantitative real-time PCR (qPCR) was performed on a StepOnePlus real-time PCR system (Applied Biosystems, ABI, CA, USA) using the SYBR Green qPCR Master Mix (ABI, CA, USA). Expression data were normalized to the expression of β-actin. The total RNA was reverse-transcribed to determine the miRNA expression, and the resulting cDNA was mixed with miRNA-specific Taqman primers (ABI, CA, USA) and Taqman Universal PCR Master Mix (ABI, CA, USA). U6 RNA was used as an endogenous control for data normalization of the miRNA level. These primers used for SYBR Green qPCR are shown in Table 1. Relative changes in expression were measured using the comparative threshold cycle (Ct) method and 2^−ΔΔCt^ as previously described; the results indicated the fold change of expression.

**Table 1:** Primer set list for qPCR

Target gene		Primer sequence 5′-3′
IRAKl	Forward	GCTCCCAGACCCATTCTGAG
	Reverse	CTCTGGGCTGGCTTGATGG
TRAF6	Forward	GCCCATGCCGTATGAAGAGA
	Reverse	ACTGAATGTGCAGGGGACTG
β-actin	Forward	CACCCGCGAGTACAACCTTC
	Reverse	CCCATACCCACCATCACACC

### Fluorescent in situ hybridization

To examine expression of miR-146a in DRG neurons, in situ hybridization was used with locked nucleic acid probes specific for miR-146a. Rats were sacrificed under anesthesia. L4-L6 DRGs were fixed by 4% paraformaldehyde. After incubated in hybridization solution at room temperature for 2 h, the sections were incubated overnight in hybridization solution with 8 ng/μL of FAM (488) labeled probes for miR-146a-5p (5′-FAM-AACCC ATGGA ATTCA GTTCT CT-FAM-3′, Wuhan Servicebio technology) at 37 °C. The sections were washed in 2 × SSC at 37 °C for 10 min and in 0.5 × SSC at room temperature for 10 min. Slides were then coverslipped with VECTASHIELD Mounting Medium with DAPI.

### Immunohistochemistry

After the rats were anesthetized with sodium pentobarbital, they were perfused transcardially with fresh 4% paraformaldehyde. L4-L6 DRGs were harvested, postfixed in 4% paraformaldehyde for 2 h, and then dehydrated in 30% sucrose overnight at 4 °C. The tissues were embedded in the optimal cutting temperature compound according to our previous studies. Frozen sections (each with 15 μm thickness) were used for immunohistochemistry analysis. The tissue sections were incubated with following primary antibodies. Then, tissue sections were incubated with the proper secondary antibodies or Alexa Fluor 594-conjugated isolectin B4 (IB4) (1:100, Invitrogen/Thermo Fisher Scientific, USA) for 1 h. Slides were then washed in PBS and coverslipped with VECTASHIELD Mounting Medium with DAPI. Table 2 lists the primary and secondary antibodies used for the immunofluorescence staining analysis.

**Table 2:** List of primary and secondary antibodies used for immunofluorescence staining

Antibody	Host	Company	Catalog ID	Dilution	Incubation conditions
IRAK1	Rabbit	Abcam	ab238	1:200	Overnight 4 °C
TRAF6	Rabbit	Abcam	ab181622	1:200	Overnight 4 °C
pNF-kB (p65)	Rabbit	Abcam	ab86299	1:200	Overnight 4 °C
CGRP	Goat	LifeSpan BioSciences	LS-C122785	1:500	Overnight 4 °C
PGP9.5	Guinea pig	Abcam	ab10410	1:100	Overnight 4 °C
Anti-rabbit IgG Alexa Fluor 488	Donkey	Jackson ImmunoRresearch	711-545-152	1:400	1 h RT
Anti-goat IgG Alexa Fluor 594	Donkey	Jackson ImmunoRresearch	705-585-147	1:400	1 h RT

### Western blot

Total proteins from rat L4-L6 DRGs or SDH were extracted with lysis buffer (CWBio, Beijing, China). Briefly, 30 μg of each sample was resolved through sodium dodecyl sulfate polyacrylamide gel electrophoresis and then transferred onto Immobilon-P polyvinylidene difluoride (GE). After blocking with 5% BSA for 1 h at room temperature, the membranes were incubated with an anti-IRAK1 antibody, anti-TRAF6 antibody, anti-pNF-κB (p65) antibody, and anti-β-actin antibody. The corresponding secondary antibodies were probed after washing the membranes. Final results were acquired using a western blot detection system (GE) with enhanced chemiluminescence reagents eECL Kit (CWBio, Beijing, China). Table 3 lists the primary and secondary antibodies used for the western blot analysis.

**Table 3:** List of primary and secondary antibodies used for western blot analysis

Antibody	Host	Company	Catalog number	Dilution	Incubation conditions
IRAK1	Rabbit	Abcam	ab238	1:1000	Overnight 4 °C
TRAF6	Rabbit	Abcam	ab181622	1:1000	Overnight 4 °C
pNF-κB (p65)	Mouse	Cell signaling technology	#13346	1:1000	Overnight 4 °C
β-actin	Mouse	ZSGB-BIO	TA-09	1:1000	Overnight 4 °C
Anti-rabbit IgG horseradish peroxidase (HRP)	Goat	ZSGB-BIO	ZDR-5306	1:3000	1 h RT
Anti-mouse IgG horseradish peroxidase (HRP)	Goat	ZSGB-BIO	ZDR-5307	1:3000	1 h RT

### Statistical analysis

Data are expressed as mean and standard errors (mean ± SEM). Statistical analyses were performed using SPSS software (vision 17.0). Differences between two groups were analyzed using Student’s *t* test. One-way ANOVA followed by Bonferroni’s post hoc tests was used to determine statistical differences among western blot and qPCR. Two-way ANOVA followed by Bonferroni’s post hoc tests was used to analyze the behavioral data. *P* < 0.05 was considered statistically significant.

## Results

### Expression level of miRNA-146a-5p is elevated in DRG and SDH neurons of rat after CCI

**Fig. 1:**
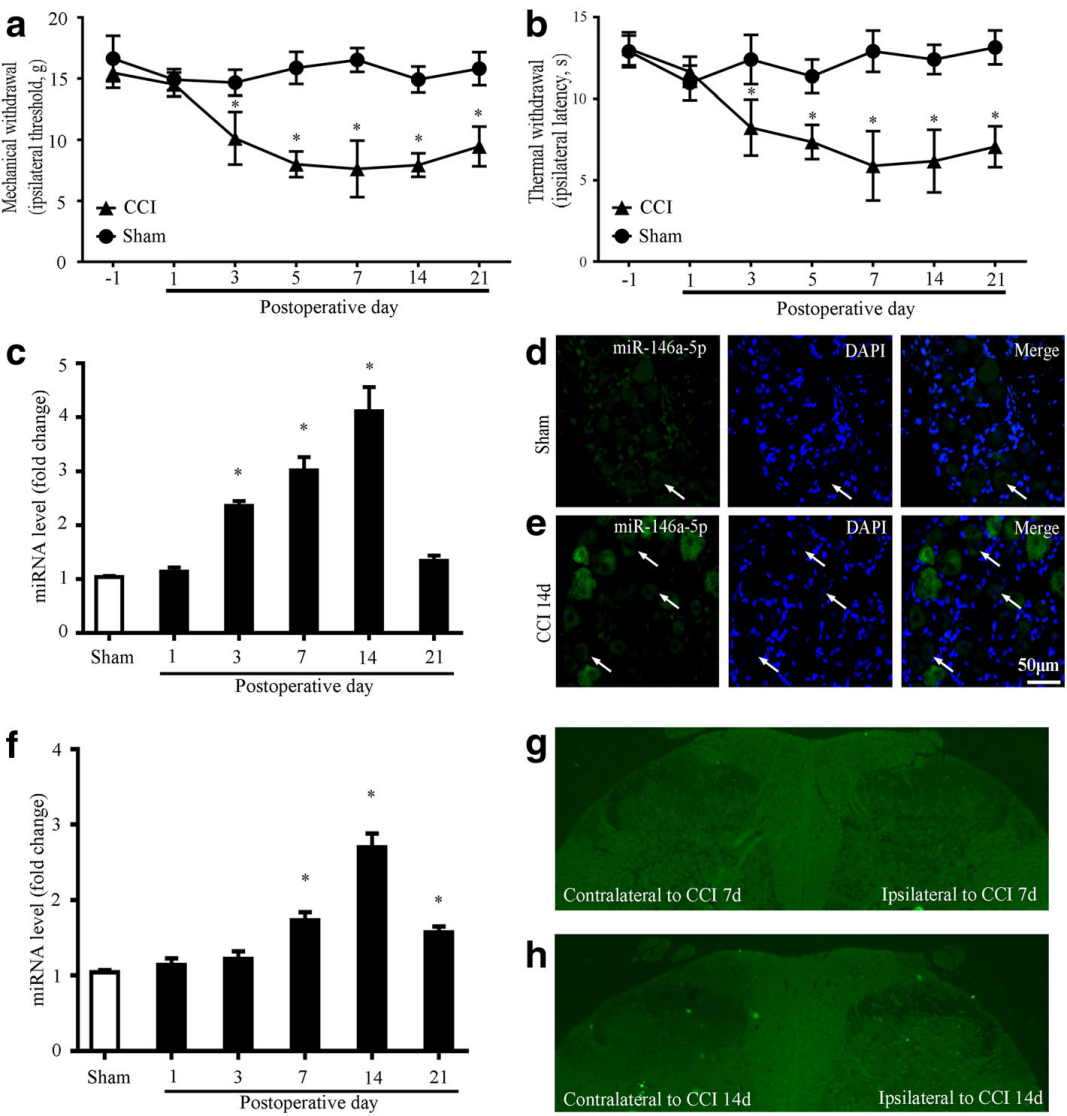
Expression of miRNA-146a-5p in rat DRG and SDH after CCI. **a**, **b** CCI-induced mechanical allodynia and thermal hyperalgesia manifested as a lowered threshold of mechanical withdrawal (**a**) and thermal withdrawal (**b**). Eight rats were included in each group. Two-way ANOVA, *P < 0.05, versus sham. **c** qPCR showing the time course for miRNA-146a-5p level in DRG (*n* = 4 in each group). One-way ANOVA, *P < 0.05, versus sham. **d**, **e** FISH showing expression and distribution of miRNA-146a-5p in rat DRGs of sham (**d**) and CCI 14 days (**e**). Scale bar 50 μm. **f** qPCR showing the time course for miRNA-146a-5p level in SDH (*n* = 4 in each group). One-way ANOVA, *P < 0.05, versus sham. **g**, **h** FISH showing expression and distribution of miRNA-146a-5p in rat SDH of CCI 7 days (**g**) and CCI 14 days (**h**)

The TIR signaling is critical for nerve injury-induced neuropathic pain generation and maintenance. Our recent results revealed that CCI increased the level of phospho-NF-kappaB of DRG. The miRNA-146a-5p is the microRNA of NF-kappaB-dependent induction. To investigate the role of miRNA-146a-5p in nerve injury-induced neuropathic pain, we used the CCI model of rat to induce neuropathic pain. Compared with sham operation rats, CCI group rats showed a rapid and persistent mechanical allodynia and thermal hyperalgesia which achieved a significant decrease in paw mechanical threshold and thermal withdrawal latency from postoperative day (POD) 3 to POD21 (Fig. 1a–b).

We first examined the expression level of miRNA-146a-5p in the rat DRG of CCI model. The qPCR results showed that miRNA-146a-5p of CCI expression was gradually, slightly, and long-lastingly increased from POD3 to POD14 and recovered to the sham level at POD21 in the DRG of rat (Fig. 1c). We further determined the cellular distribution of miRNA-146a-5p in the DRG of CCI operation with fluorescent in situ hybridization (FISH). We found that the miRNA-146a-5p was distributed in large-sized, medium-sized, and small neurons, but increased staining miRNA-146a-5p was distributed in the DRG of CCI (Fig. 1d–e). In the SDH, our qPCR analysis showed that the CCI operation produced increased from POD7 to PDO21 in the miRNA-146a-5p level (Fig. 1f). FISH results showed that the increased miRNA-146a-5p was distributed in the SDH of the spinal cord (Fig. 1g–h).

### CCI increases level of IRAK1 and TRAF6 in DRG neurons of rat

**Fig. 2:**
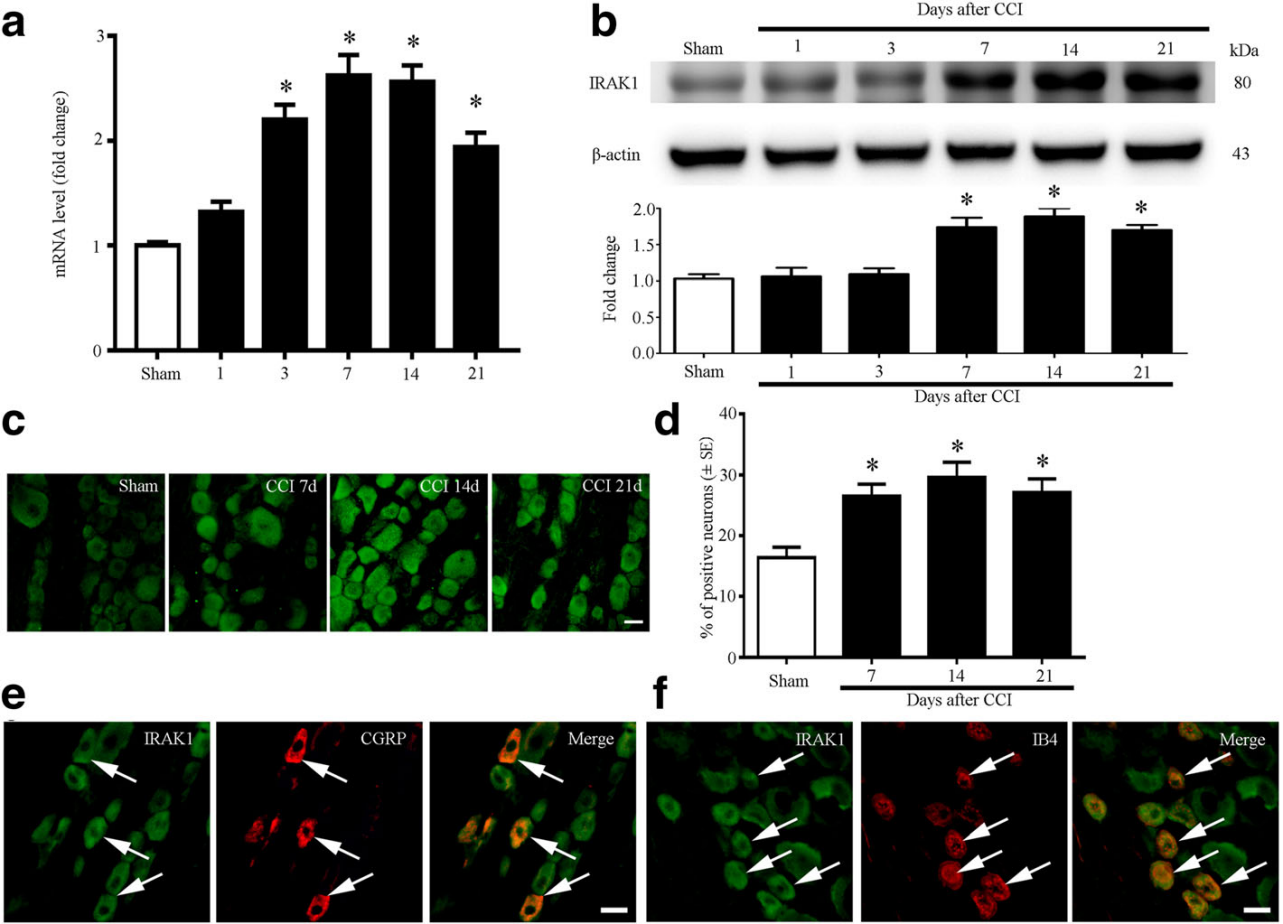
Expression and cellular distributions of IRAK1 in rat DRG after CCI. **a** qPCR showing the time course for IRAK1 level in DRG (*n* = 4 in each group). One-way ANOVA, *P < 0.05, versus sham. **b** Western blot analyzes the time course for IRAK1 expression in DRG. Representative bands are shown on the top; data summary is shown on the bottom. One-way ANOVA, *P < 0.05, versus sham. **c** Immunofluorescence showing cellular distribution of IRAK1 in rat DRGs of sham, CCI 7-day, CCI 14-day, and CCI 21-day group. **d** Quantification analysis from immunofluorescence staining results of IRAK1 in DRG neurons. Percentage of IRAK1-positive neurons in DRGs of sham, CCI (7 days), CCI (14 days), and CCI (21 days) rats. *n* = 4 in each group, One-way ANOVA, *P < 0.05, versus sham. **e** Double immunostaining showing co-expression of IRAK1 (green) with CGRP (red) in rat DRG of CCI 14 day. **f** Double immunostaining showing co-expression of IRAK1 (green) with IB4 (red) in rat DRG of CCI 14 days. Scale bar 25 μm in **c**, **e**, and **f**

IRAK1 is recruited by MyD88 and initiate TIR signaling. Our published results revealed that MyD88 protein was increased in the DRG of CCI rats. To examine IRAK1 expression level in the DRG of CCI rats, qPCR results revealed that mRNA level of IRAK1 was continuously increased from POD3 to POD21 in DRG after CCI (Fig. 2a). Similar with the mRNA, western blot analysis showed that the protein expression of IRAK1 was significantly increased in POD7 and peaked in POD14 in DRG after CCI (Fig. 2b). To check the cellular distribution of IRAK1 in the DRG, we did stain of immunofluorescence of IRAK1. Our results showed that IRAK1 immunoreactivity (IRAK1-IR) cells were distributed in the three-size category of DRG neurons of sham and CCI group. We found the percentages of IRAK1-IR neurons of DRG were significantly increased after CCI operation (Fig. 2c–d and Additional file 1: Figure S1). We also found IRAK1 was expressed in calcitonin gene-related peptide (CGRP)-positive neurons and IB4-positive neurons in the DRG (Fig. 2e–f).

**Fig. 3:**
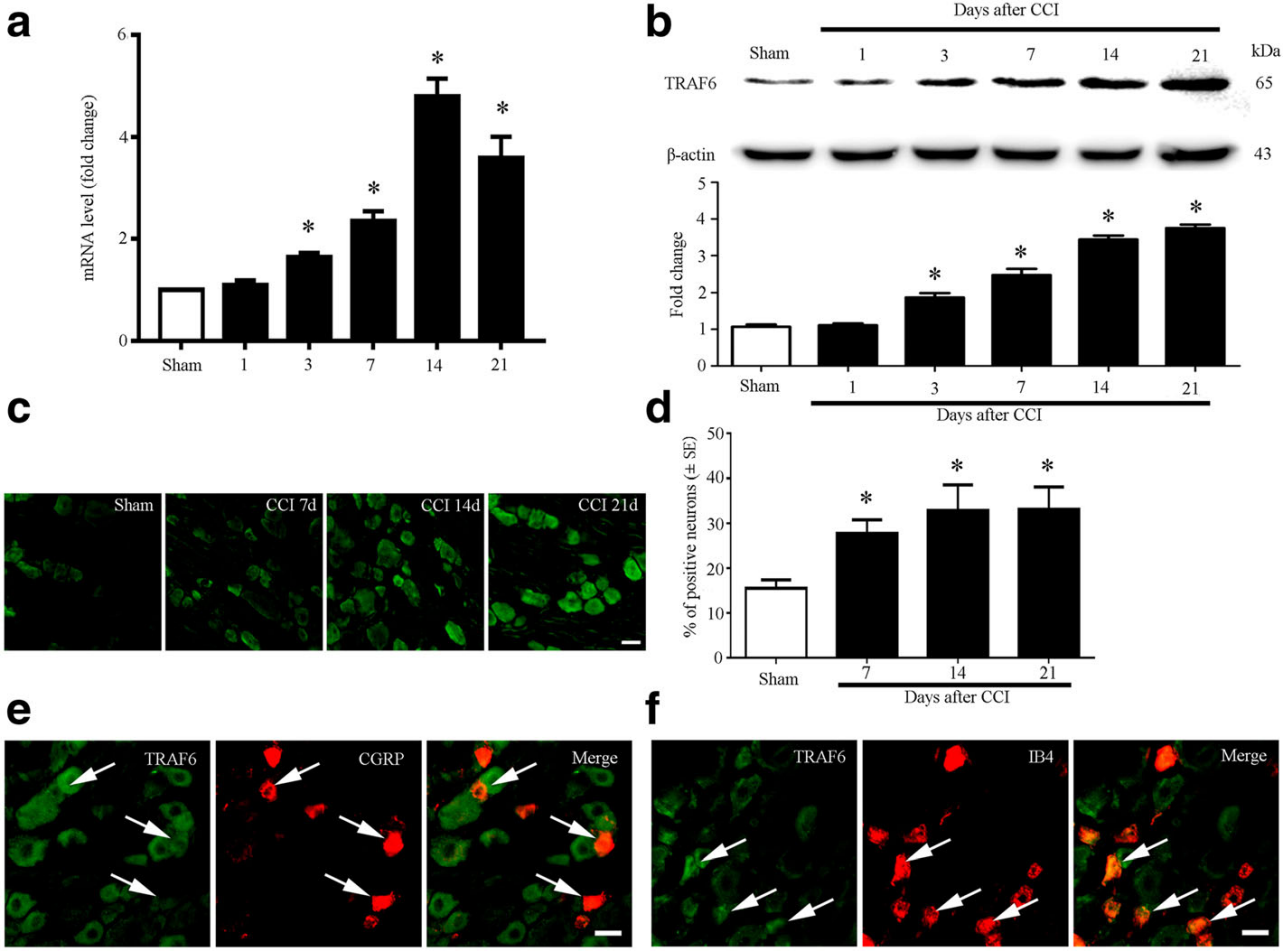
Expression and cellular distributions of TRAF6 in rat DRG after CCI. **a** qPCR showing the time course for TRAF6 level in DRG (*n* = 4 in each group). One-way ANOVA, *P < 0.05, versus sham. **b** Western blot analyzes the time course for TRAF6 expression in DRG. **c** Cellular distribution of TRAF6 in rat DRGs of sham, CCI 7 days, CCI 14 days, and CCI 21 day. **d** Percentage of TRAF6-positive neurons in DRGs of sham, CCI (7 days), CCI (14 days), and CCI (21 days) rats. *n* = 4 in each group, One-way ANOVA, *P < 0.05, versus sham. **e** Double immunostaining showing co-expression of TRAF6 (green) with CGRP (red) in rat DRG of CCI 14 day. **f** Double immunostaining showing co-expression of TRAF6 (green) with IB4 (red) in rat DRG of CCI 14 days. Scale bar 25 μm in **c**, **e**, and **f**

The mRNA level of TRAF6 was also detected through qPCR. We found the mRNA level of TRAF6 was increased in POD3, peaked in POD14, and continuously increased to POD21 (Fig. 3a). We then examined the protein level of TRAF6 in rat DRG. western blot results showed that the expression of TRAF6 was started increasing in POD3 and maintained in POD21 after CCI operation compared with the sham operation (Fig. 3b).

We also examined the distribution of TRAF6 in DRG. Immunofluorescence results showed that TRAF6 immunoreactivity (TRAF6-IR) cells were distributed in the three-size category of DRG neurons in the CCI group. CCI induced a marked increase of TRAF6-IR in the ipsilateral side of the DRG at POD7, POD14, and POD21 (Fig. 3c, d and Additional file 1: Figure S2). To check whether TRAF6 is expressed in the nociceptive neuron, we did double immunofluorescence of TRAF6 with nociceptive neuronal markers CGRP and IB4. We found that TRAF6-IR was colocalized with CGRP and IB4 (Fig. 3e, f).

### Intrathecal injection miRNA-146a-5p agomir and miRNA-146a-5p antagomir regulate miRNA-146a-5p expression levels in DRG and SDH

**Fig. 4:**
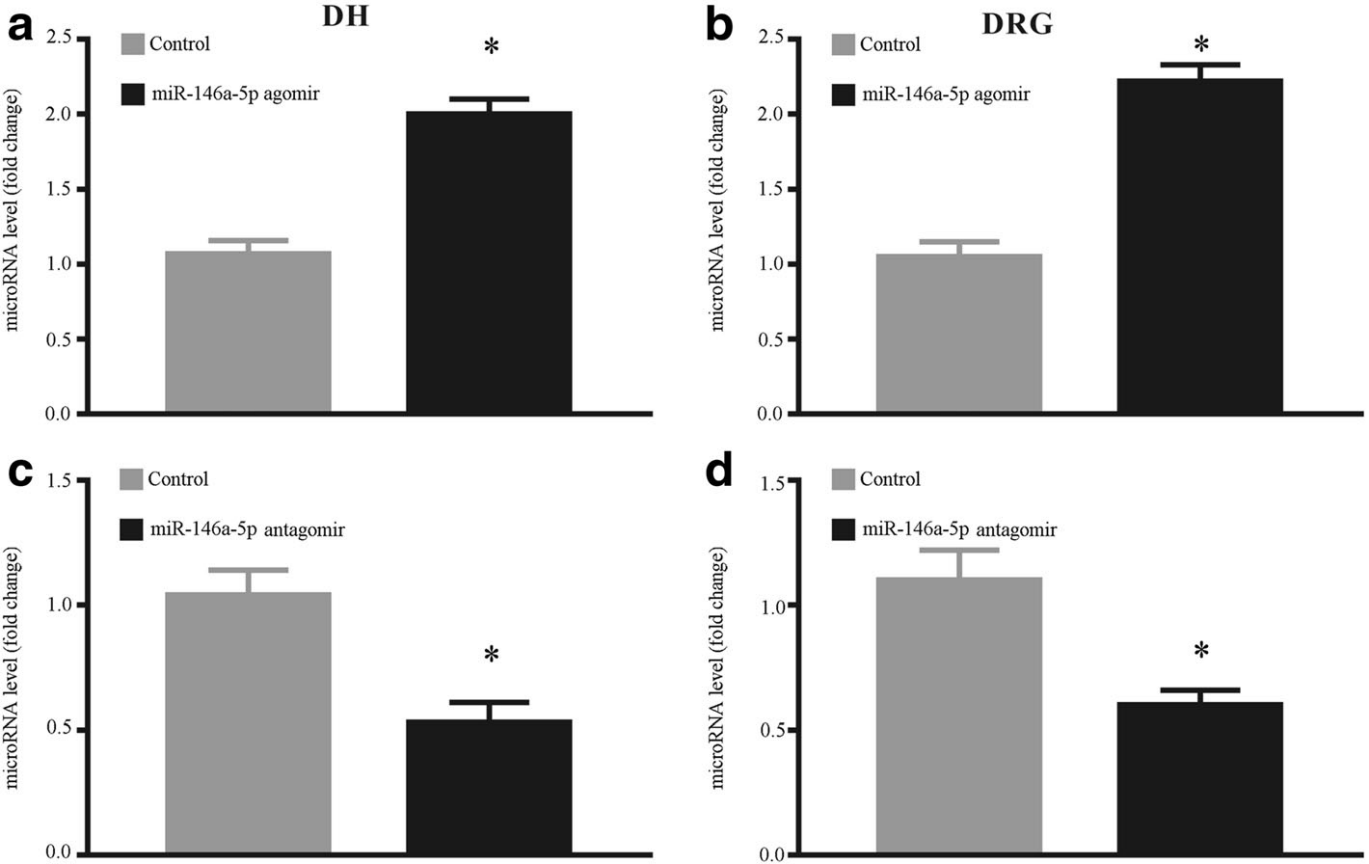
Expression of miRNA-146a-5p in SDH and DRG of rat with intrathecal administration of miRNA-146a-5p agomir or miRNA-146a-5p antagomir. **a**, **b** qPCR showed intrathecal administration of miRNA-146a-5p agomir upregulated the RNA level of miRNA-146a-5p in SDH (**a**) and DRG (**b**). **c**, **d** qPCR showed intrathecal administration of miRNA-146a-5p antagomir reduced the RNA level of miRNA-146a-5p in SDH (**c**) and DRG (**d**)

Agomir is a double-stranded miRNA that is specially marked and chemically modified to regulate the biology of the target gene. To evaluate the effect of miRNA-146a-5p agomir for increasing the miR146a-5p level, we intrathecally injected miRNA-146a-5p agomir in native rats. The expression level of miRNA-146a-5p in SDH and DRG was accessed by qPCR after intrathecally injected miRNA-146a-5p agomir. qPCR results indicated that the expression level of miRNA-146a-5p was increased in L4-L6 DRGs and SDH of rats after intrathecally injected with miRNA-146a-5p agomir compared with agomir control rats (Fig. 4a, b). We also evaluated the effect of miRNA-146a-5p antagomir on the expression of miR-146a-5p and found that compared to the antagomir control group, miRNA-146a-5p antagomir decreased the expression level of miR-146a-5p (Fig. 4c, d).

### miRNA-146a-5p agomir relieves CCI-induced neuropathic pain

**Fig. 5:**
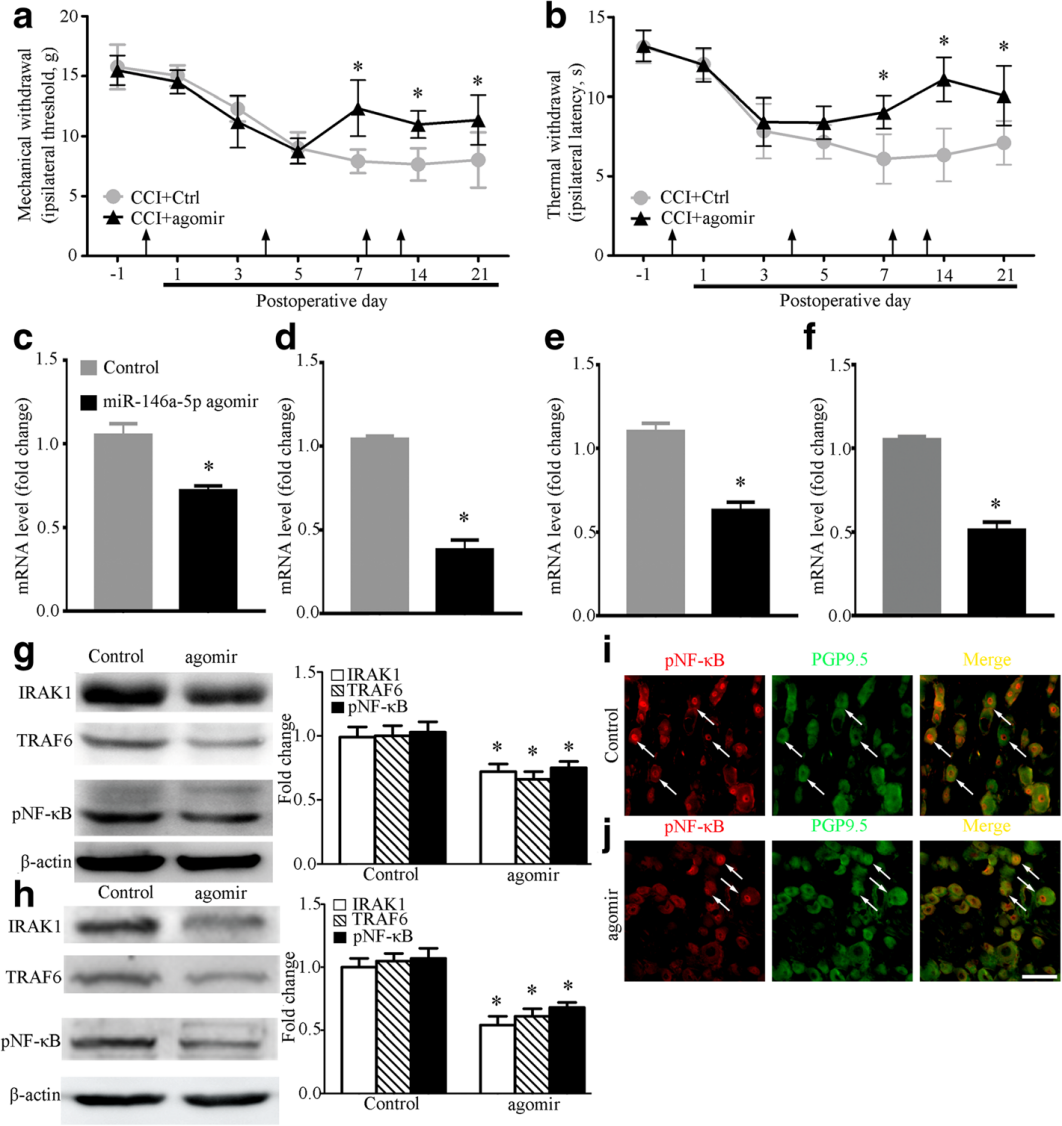
miRNA-146a-5p attenuated neuropathic pain and decreased IRAK1 and TRAF6 expression in rat DRG and SDH after CCI. **a**, **b** Intrathecal injection of miRNA-146a-5p agomir attenuated CCI-induced mechanical allodynia (**a**) and thermal hyperalgesia (**b**). Each administration is indicated by an arrow on 0, 4, 8, and 12 days after CCI operation. Two-way ANOVA, *P < 0.05 versus CCI + Ctrl. miRNA-146a-5p agomir was administrated i.t. in a volume of 20 μL. The negative miRNA agomir was used in the control group. Eight rats were included in each group. **c**, **d** qPCR showing mRNA level of IRAK1 mRNA in SDH (**c**) and DRG (**d**) after miRNA-146a-5p agomir administration. **e**, **f** qPCR showing mRNA level of TRAF6 in SDH (**e**) and DRG (**f**) after miRNA-146a-5p agomir administration. *n* = 4 in each group of **c**, **f**, Student’s *t* test, *P < 0.05, versus control. **g**, **h** Western blot showing protein level of IRAK1, TRAF6, and pNF-κB (p65) in SDH (**g**) and DRG (**h**) after miRNA-146a-5p agomir administration. Data summary is shown on the right. *n* = 4 in each group, Student’s *t* test, *P < 0.05 versus sham. **i**, **j** Immunofluorescence showing nuclear translocation of pNF-κB (p65) in CCI control group (**i**) and CCI agomir group (**j**). Scale bar 50 μm (**i**, **j**)

Compared with CCI rats that were injected intrathecally with agomir control, injection intrathecally with miRNA-146a-5p agomir significantly attenuated CCI-induced neuropathic pain from POD7 until POD21 (Fig. 5a, b). Subsequently, our qPCR results found that injection intrathecally with miRNA-146a-5p agomir significantly decreased the IRAK1 mRNA level in SDH and DRG of rats with CCI POD14 (Fig. 5c, d). In addition, we also found that the TRAF6 mRNA level in SDH and DRG were decreased in CCI rats with intrathecal miRNA-146a-5p agomir (Fig. 5e, f). We further investigated the potential effects of miRNA-146a-5p agomir on IRAK1 and TRAF6 protein level in DRG and SDH at 14 days after CCI. In comparison with the rats in the CCI rats injected with negative control, western blot showed that miRNA-146a-5p agomir significantly decreased the protein level of IRAK1 and TRAF6 in DRG and SDH (Fig. 5g, h). Meanwhile, our western blot results also found that intrathecal miRNA-146a-5p agomir decreased the phosphorylation level of pNF-κB (p65) protein in CCI rats (Fig. 5g, h). Our double immunofluorescence results showed CCI induced nuclear translocation of pNF-κB (p65) in DRG neurons of rats which were intrathecally injected with miRNA-146a-5p agomir or control agomir (Fig. 5i, j).

### miRNA-146a-5p antagomir aggravate neuropathic pain of rats after CCI

**Fig. 6:**
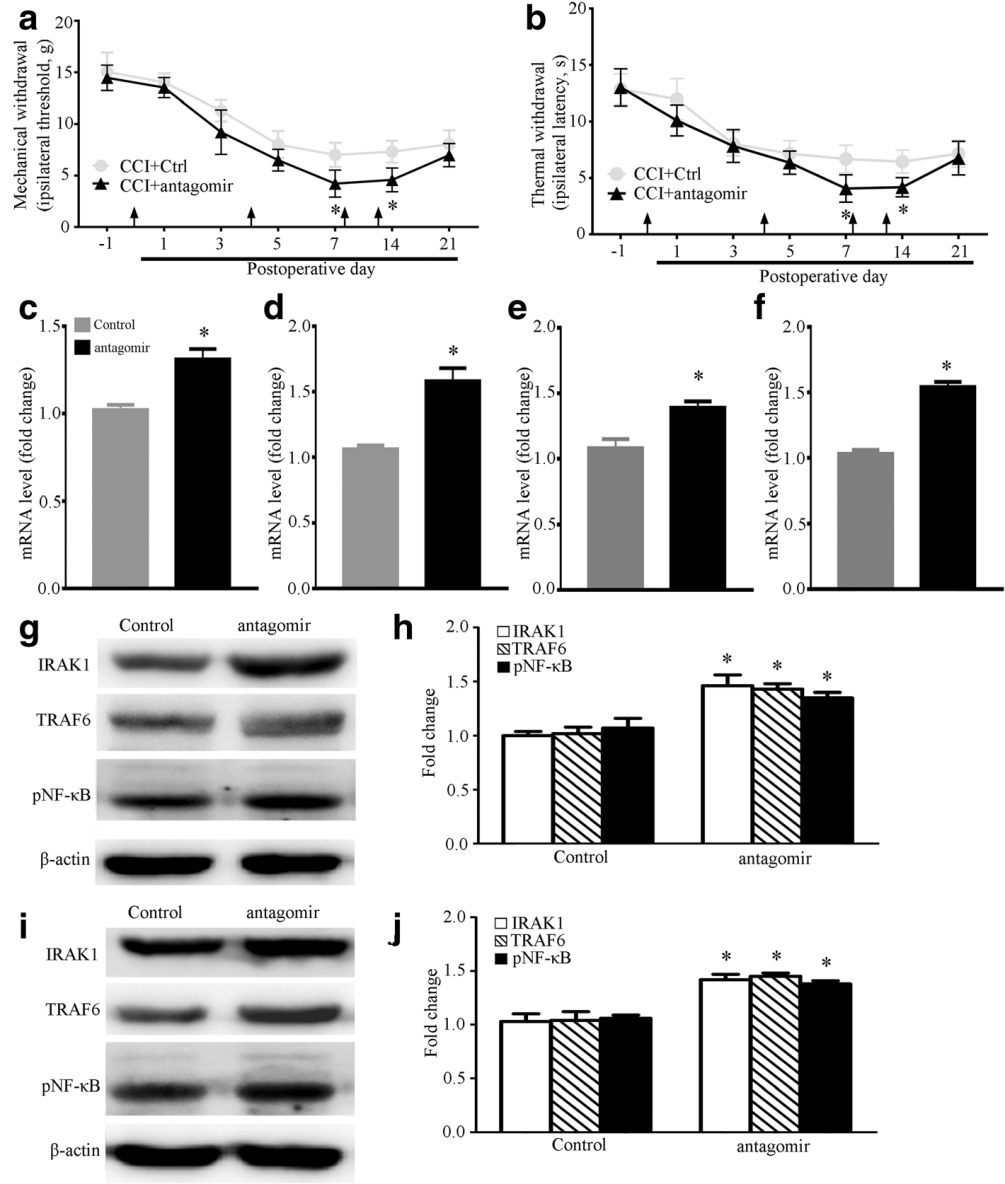
Inhibition of miRNA-146a-5p leads to aggravated neuropathic pain and increased IRAK1 and TRAF6 expression in rat DRG and SDH after CCI. **a**, **b** Intrathecal injection of miRNA-146a-5p antagomir aggravated CCI-induced mechanical allodynia (**a**) and thermal hyperalgesia (**b**). Each administration is indicated by an arrow on 0, 4, 8, and 12 days after CCI operation. Two-way ANOVA, *P < 0.05, versus CCI + Ctrl. miRNA-146a-5p antagomir was administrated i.t. in a volume of 20 μL. The negative miRNA antagomir was used in the control group. Eight rats were included in each group. **c**, **d** qPCR showing mRNA level of IRAK1 mRNA in SDH (**c**) and DRG (**d**) after miRNA-146a-5p antagomir administration. **e**, **f** qPCR showing mRNA level of TRAF6 in SDH (**e**) and DRG (**f**) after miRNA-146a-5p antagomir administration. *n* = 4 in each group of **c**–**f**, Student’s *t* test, *P < 0.05, versus control. **g–j** Western blot showing protein level of IRAK1, TRAF6, and pNF-κB (p65) in SDH (**g**, **h**) and DRG (**i**, **j**) after miRNA-146a-5p antagomir administration. Data summary of western blot results in SDH (**h**) and DRG (**j**) is shown on the right. *n* = 4 in each group, Student’s *t* test, *P < 0.05 versus sham

We further determined the effect of miR-146a-5p antagomir on CCI rats. Compared with CCI rats that were injected intrathecally antagomir control, injection intrathecally with miR-146a-5p antagomir significantly aggravated CCI-induced neuropathic pain from POD7 to POD21 (Fig. 6a, b). Our qPCR results suggested that miR-146a-5p antagomir increased the mRNA level of IRAK1 and TRAF6 in DRG and SDH at 14 days after CCI compared with CCI rats which were intrathecally injected with antagomir control (Fig. 6c, f). Western blot results showed that miR-146a-5p antagomir increased the protein level of IRAK1 and TRAF6 in DRG and SDH at CCI POD14 (Fig. 6g–j). We also found intrathecal miR-146a-5p antagomir increased the phosphorylation level of pNF-κB (p65) protein in CCI rats (Fig. 6g–j).

## Discussion

Neuropathic pain is a common type of chronic pain that affects the life quality of patients. The exact molecular mechanism of neuropathic pain has not been fully elucidated. Several rat models with partial injury to peripheral nerves have been used to investigate the possible mechanisms. CCI is a commonly used model to mimic the pathophysiological progress of chronic neuropathic pain. In this study, the role of miRNA-146a-5p in the pathophysiological mechanism of neuropathic pain was investigated. Our research successfully established a CCI rat model and found that the mechanical PWT and thermal PWL in the CCI group were significantly lower than those in the sham group. The CCI rats showed allodynia and hyperalgesia, which are precise clinical characteristics of neuropathic pain. Our results demonstrated a significant increase in the miRNA-146a-5p level in DRG and SDH of rats suffering from neuropathic pain and a considerable increase in the expression of IRAK1 and TRAF6. Our findings are consistent with other studies that used other pain models, in which miRNA-146a-5p, TRAF6, or IRAK1 are strongly upregulated in the SDH of pain models [26, 30, 31].

Several reports showed some miRNAs participate in the development of neuropathic pain and affect neuropathic pain by regulating protein level in the pain progress [19, 32–34]. The proposed possible mechanism indicates that peripheral stimuli from inflammation or nerve injury can induce the secretion of inflammatory mediators and thus change the miRNA expression in DRG or SDH. miRNA-146a-5p, a member of the miRNA family, is involved in immune responses, cell proliferation, and inflammation [18, 35]. miRNA-146a-5p is related to pain-related pathophysiology of osteoarthritis. The variable expression of miRNA-146a-5p in the spinal cord and DRG contributes to osteoarthritic pain in the knee joint [25, 31].

As a critical innate immune receptor, TLRs is activated in neuropathic pain, and its deficiency protects against neuropathic pain. The activation of the TLRs signaling on cells in the peripheral or central nervous system, particularly the glia cell and DRG neuron, contributes to neuropathic pain [5–8, 15, 36, 37]. Activated TLR4 initiates transmembrane signaling cascades that trigger intracellular mediators [13–16]. In this pathway, the activation of IRAK1 and TRAF6 leads to the nuclear translocation of the transcription factor NF-κB, resulting in the production of proinflammatory cytokines, such as IL-6 and TNF-α. Meanwhile, the activation of NF-κB can induce miRNA-146a-5p [17]. miRNA-146a-5p that is NF-κB-dependent microRNA plays a key role in the regulation of TIR signaling through its target molecules, namely, TRAF6 and IRAK1, which are two important protein kinase in the TIR signaling pathway [17, 22, 24]. We demonstrated that over-expression of miRNA-146a-5p protects rats against neuropathic pain after CCI operation by negatively regulating the expression level of IRAK1 and TRAF6.

To further determine the role of miRNA-146a-5p in the CCI-induced neuropathic pain, we found CCI rats which were intrathecally injected with miRNA-146a-5p antagonist; miRNA-146a-5p antagomir suffer from aggravated neuropathic pain. Intrathecal injection with miRNA-146a-5p antagomir elevated the level of IRAK1 and TRAF6 of CCI rats. Our finding is consistent with recent studies, in which miRNA-146a-5p negatively regulates the TIR signaling pathway by targeting IRAK1 and TRAF6. Several studies suggested that miRNA-146a-5p may negatively regulate the LPS-induced TLR signaling through downregulation of IRAK1 and TRAF6 by binding to the 3′UTR of their mRNAs [17, 38]. Previous studies also confirmed that miRNA-146a-5p-deficient mice exhibit a considerable increase in IRAK1 and TRAF6 protein level and are hypersensitive to LPS [23, 39]. However, whether miR-146a-null mice are sensitive to neuropathic pain must be further confirmed. In our research, we demonstrated that miRNA-146a-5p antagomir increased the phosphorylation level of NF-κB (p65). That indicated downregulation of miRNA-146a-5p may result in the over-responsiveness of TIR signaling pathway. By contrast, the over-expression of miRNA-146a-5p contributed to the lower level of phosphorylation for NF-κB (p65). In this study, we did not study the expression of miRNA-146a-5p and its targets in the brain after neuropathic pain. The expression of miRNA-146a-5p on the brain can possibly regulate neuropathic pain, yet this hypothesis must be further confirmed.

## Conclusions

In this study, we demonstrated that neuropathic pain was associated with miRNA-146a-5p. The therapeutic approaches using miRNA-146a-5p agomir could relieve neuropathic pain in rat models of CCI. The mechanism may involve the regulation of the TIR signaling pathway by directly suppressing its target, IRAK1 and TRAF6. The administration of miRNA-146a-5p or its inducers can be used as a promising therapy to relieve neuropathic pain.

## Additional file

Additional file 1: **Figure S1.** Cellular distribution of IRAK1 in DRGs. Figure S2 Cellular distribution of TRAF6 in DRGs. (DOC 925 kb)

## Abbreviations

CCI: Chronic constriction injury
CGRP: Calcitonin gene-related peptide
DRG: Dorsal root ganglion
FISH: Fluorescent in situ hybridization
IB4: Isolectin B4
IRAK1: Interleukin-1 receptor-associated kinase 1
MyD88: Myeloid differentiation primary response protein 88
NF-κB: Transcription factors of the nuclear factor kappa B
POD: Postoperative day
PWL: Paw withdrawal latency
PWT: Paw withdrawal threshold
SDH: Spinal dorsal horn
TIR: Toll/interleukin-1 receptor
TLR: Toll-like receptor
TRAF6: TNF receptor-associated factors 6
