# Supplementary material for: Chronic constriction injury-induced microRNA-146a-5p alleviates neuropathic pain through suppression of IRAK1/TRAF6 signaling pathway
Source: J Neuroinflammation. 2018 Jun 9;15:179. doi: 10.1186/s12974-018-1215-4 (PMC5994250; doi:10.1186/s12974-018-1215-4)
Supplement: Supplementary file 1 — Figure S1. Cellular distribution of IRAK1 in DRGs. Figure S2 Cellular distribution of TRAF6 in DRGs. (DOC 925 kb) [file 12974_2018_1215_MOESM1_ESM.doc]

**Additional file 1**


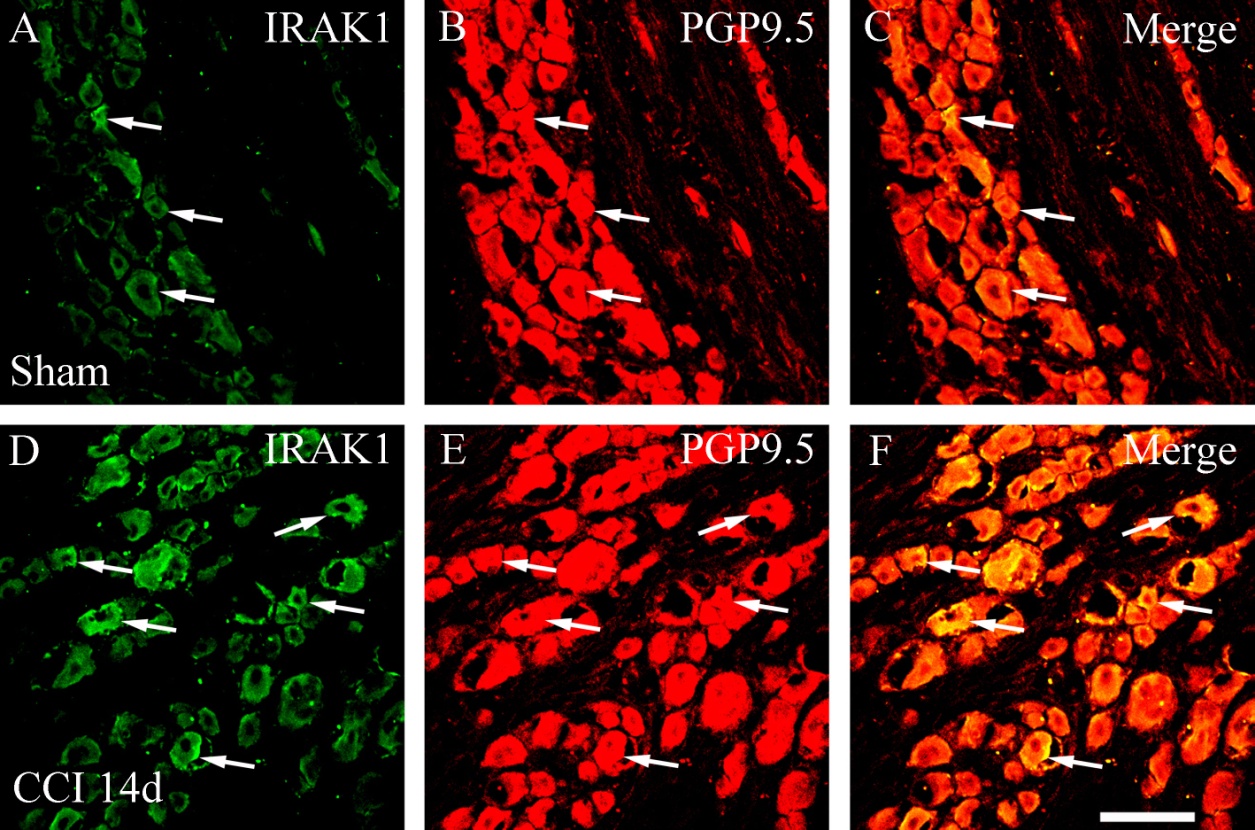


**Figure S1. Cellular distribution of IRAK1 in DRGs. A-C.** Double immunostaining shows IRAK1 (A, green) was co-expressed in the neurons (B, red, PGP9.5 protein is the neuronal marker) of sham rats. **D-F.** Double immunostaining shows IRAK1 (D, green) was co-expressed in the neurons (E, red) of CCI 14day rats. Bar scale: 100μm.


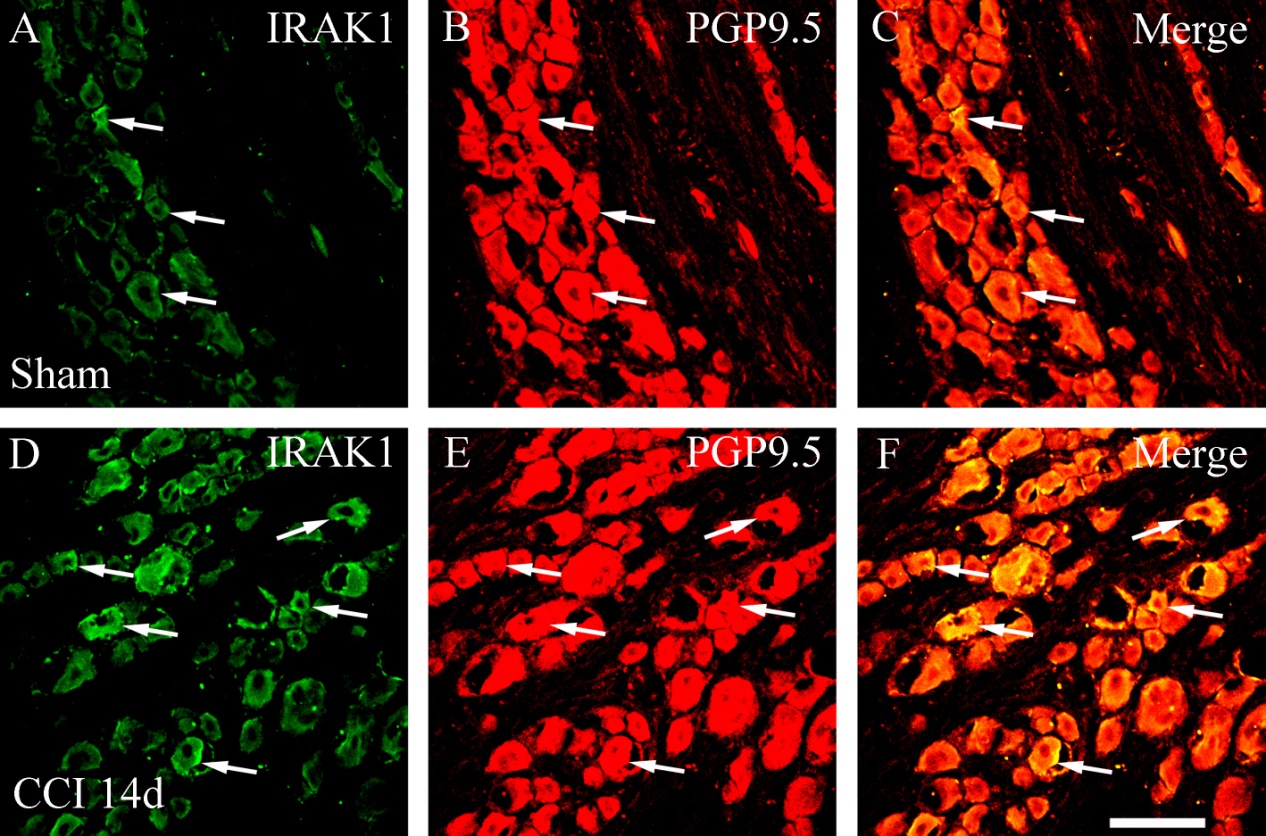


**Figure S2. Cellular distribution of TRAF6 in DRGs. A-C.** Double immunostaining shows TRAF6 (A, green) was co-expressed in the neurons (B, red, PGP9.5 protein is the neuronal marker) of sham rats. **D-F.** Double immunostaining shows TRAF6 (D, green) was co-expressed in the neurons (E, red) of CCI 14day rats. Bar scale: 100μm.
